# Acupuncture for Parkinson’s disease: targeting programmed cell death mechanisms and therapeutic prospects

**DOI:** 10.3389/fneur.2026.1776605

**Published:** 2026-04-02

**Authors:** Dandan Wang, Shichang Liu, Binbin Ren

**Affiliations:** 1 Rehabilitation Medicine College, Henan University of Chinese Medicine, Zhengzhou, China; 2 Department of Rehabilitation, The First Affiliated Hospital of Henan University of Chinese Medicine, Zhengzhou, China; 3 Zhengzhou First People's Hospital, Zhengzhou, China

**Keywords:** acupuncture, apoptosis, autophagy, ferroptosis, necroptosis, Parkinson’s disease, programmed cell death, pyroptosis

## Abstract

As the population ages, Parkinson’s disease (PD) has become a prevalent neurodegenerative disorder, posing a significant threat to the health and quality of life of the elderly. The core pathological features include progressive loss of dopaminergic neurons in the substantia nigra pars compacta and the formation of Lewy bodies, which result from aberrant aggregation of *α*-synuclein (α-syn). The pathogenesis of PD involves multi-level, cross-system cellular mechanisms. Recent evidence reveals that classical forms of programmed cell death (PCD)—including autophagy, apoptosis, pyroptosis, necroptosis, and ferroptosis—interact through key nodes such as neuroinflammation, mitochondrial dysfunction, and oxidative stress. This interplay drives dopaminergic neuron death and α-syn aggregation, thereby accelerating PD progression. Acupuncture has emerged as a prominent non-pharmacological therapeutic strategy for PD, showing beneficial effects by targeting multiple PCD pathways. This review systematically delineates the roles of autophagy, apoptosis, pyroptosis, necroptosis, and ferroptosis in PD pathogenesis. Furthermore, it identifies key molecular mediators and physiological outcomes through which acupuncture ameliorates PD by regulating PCD-related signaling, thereby providing a mechanistic rationale for the development of targeted interventions.

## 1 Introduction

Parkinson’s disease (PD), the second most common neurodegenerative disorder after Alzheimer’s disease, is closely associated with aging. Its core pathological features include the progressive loss of dopaminergic neurons in the substantia nigra pars compacta and the formation of Lewy bodies, which results from the abnormal aggregation of α-syn (1). The pathogenesis of PD is complex, involving a multifactorial interplay of mechanisms such as neuroinflammation, mitochondrial dysfunction, and oxidative stress (2). Accumulating evidence indicates that dysregulated programmed cell death (PCD) is a pivotal mechanism driving dopaminergic neuronal loss and α-syn pathology, thereby accelerating PD progression. PCD encompasses several distinct pathways, including autophagy, apoptosis, pyroptosis, necroptosis, and ferroptosis. Current clinical treatments for PD, primarily levodopa-based symptomatic therapies, fail to halt disease progression and are often associated with significant side effects (2). Notably, research suggests that acupuncture may exert neuroprotective effects by modulating the PCD network through multiple targets—for instance, by enhancing autophagy, suppressing excessive apoptosis, and regulating ferroptosis. This review systematically elucidates the mechanisms of key PCD pathways (autophagy, apoptosis, pyroptosis, necroptosis, and ferroptosis) in PD pathogenesis and summarizes the therapeutic potential of acupuncture in regulating these processes. The aim is to provide a theoretical foundation for developing precise acupuncture strategies targeting PCD in PD management.

## 2 Materials and methods

### 2.1 Data sources

#### 2.1.1 Search execution

The initial literature search was conducted by the first author in October 2025.

#### 2.1.2 Search period

All databases were searched from their inception until 2025, with particular emphasis on publications from the preceding 5 years.

#### 2.1.3 Databases

The electronic databases searched included PubMed, Web of Science, and the China National Knowledge Infrastructure (CNKI).

#### 2.1.4 Search terms

English search terms comprised “Parkinson’s disease,” “programmed cell death,” “acupuncture,” “autophagy,” “apoptosis,” “pyroptosis,” “necroptosis,” and “ferroptosis.” Corresponding Chinese terms were also used.

#### 2.1.5 Literature types

The search aimed to identify relevant reviews, experimental research articles, meta-analyses, and related publication types.

#### 2.1.6 Search strategy

**Figure 1:**
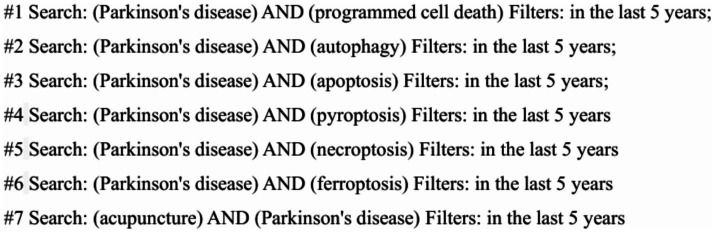
PubMed database search strategy.

The specific search strategy is detailed using PubMed as an example in Figure 1.

#### 2.1.7 Search yield

The initial database search returned approximately 4,915 records.

### 2.2 Eligibility criteria

#### 2.2.1 Inclusion criteria

Studies were included if they met the following criteria: (1) focused on PD pathophysiology or models; (2) investigated mechanisms of PCD; (3) explored the relationship between PCD and PD pathogenesis; (4) examined acupuncture or related techniques as an intervention for PD, with a reported effect on PCD pathways.

#### 2.2.2 Exclusion criteria

Studies were excluded for the following reasons: (1) lack of methodological rigor; (2) publication date prior to 1990, unless deemed a seminal work; (3) content irrelevant to the scope of this review; (4) duplicate reports of the same study data.

### 2.3 Literature screening and quality assessment

**Figure 2:**
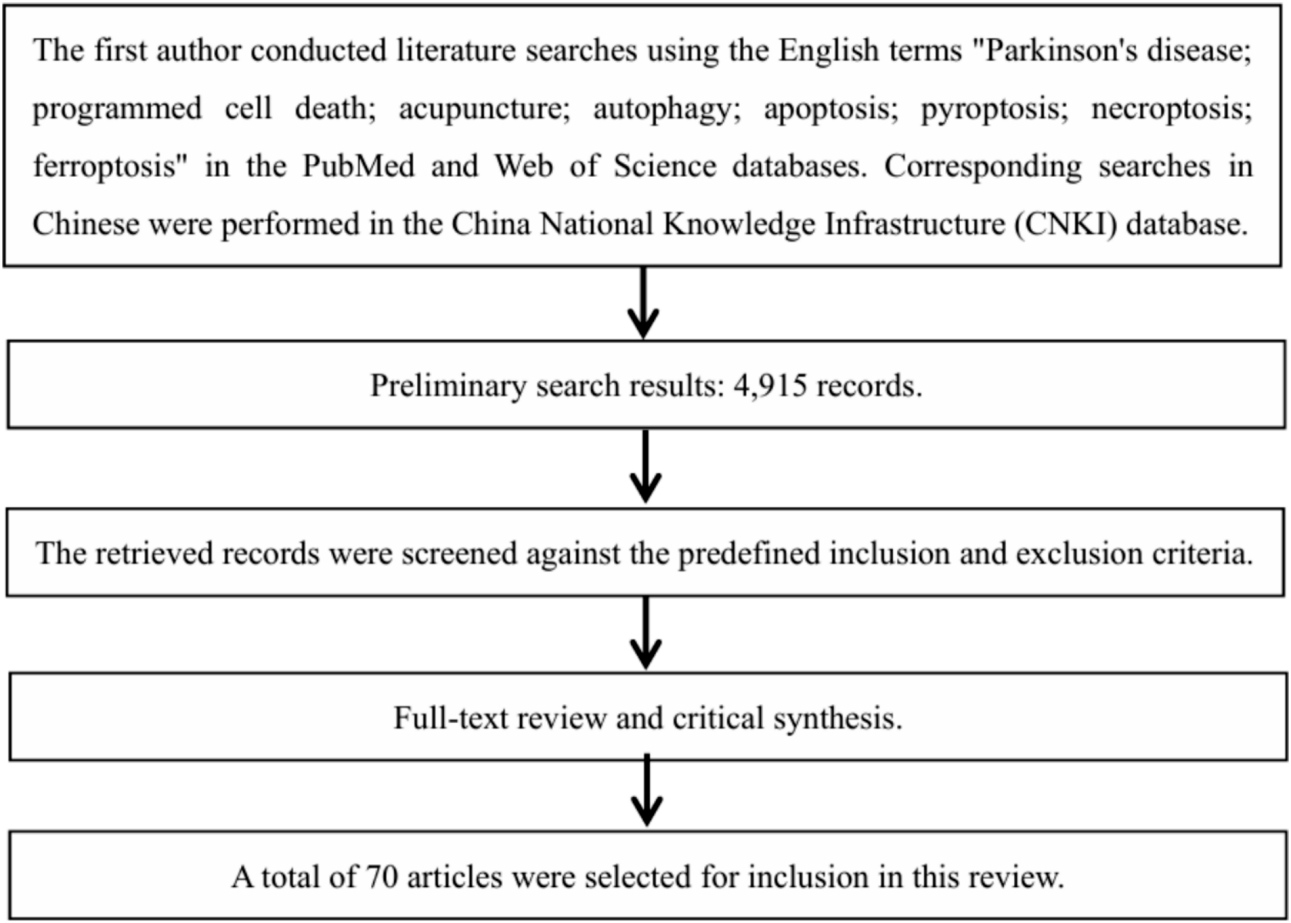
Literature screening flowchart.

A two-stage screening protocol was employed. First, titles and abstracts were reviewed against the eligibility criteria. Records that were clearly irrelevant, of low methodological quality, or outside the defined scope were excluded. Second, the full texts of the remaining records were retrieved and subjected to a detailed eligibility assessment. Following this process, a final set of 70 articles that satisfied all inclusion criteria were selected for in-depth analysis and synthesis, as summarized in the PRISMA flow diagram (Figure 2).

## 3 Mechanism of PCD in PD

### 3.1 Autophagy

Autophagy is a fundamental cellular degradation and recycling process essential for maintaining intracellular homeostasis. It specifically targets abnormal protein aggregates, damaged organelles, and surplus proteins for disposal. This process occurs mainly through three pathways: macroautophagy, microautophagy, and chaperone-mediated autophagy (CMA), with macroautophagy being the predominant form (3).

The pathology of PD is characterized by the progressive loss of dopaminergic neurons in the substantia nigra pars compacta and the formation of Lewy bodies, which are primarily composed of aggregated α-syn. The intracellular accumulation, impaired degradation, and intercellular propagation of α-syn have been identified as critical drivers of PD progression (4, 5)^.^

Impaired autophagy disrupts cellular homeostasis, directly hindering α-syn clearance, promoting its aberrant aggregation, and inducing cytotoxicity, thereby contributing to PD pathogenesis (4). Substantial evidence indicates that mutations in several PD-associated genes (e.g., LRRK2, GBA, PINK1, PRKN, *DJ-1*) and the expression of risk alleles such as APOE4 exert their pathological effects by disrupting autophagy-related pathways, notably the autophagy-lysosomal pathway and mitophagy (3).

A bidirectional regulatory relationship exists between α-syn and the autophagy system. Under pathological conditions, abnormally aggregated α-syn can significantly suppress autophagic activity through multiple mechanisms, including interfering with the Bcl2-BECN1 complex, inhibiting Rab1a function, and disrupting lysosomal homeostasis. Conversely, under physiological conditions, the autophagy-lysosomal pathway (ALP) is a crucial mechanism for clearing α-syn aggregates. Compromise of the ALP exacerbates intracellular α-syn accumulation and facilitates its intercellular spread (4).

In summary, autophagy plays a central regulatory role in maintaining α-syn metabolic balance and is integral to PD pathology. Targeted modulation of autophagy, therefore, holds broad therapeutic potential for the prevention and treatment of PD.

### 3.2 Apoptosis

Apoptosis is a form of PCD characterized by its non-inflammatory nature. It enables precise and orderly cellular responses to internal and external stresses, leading to controlled cell degradation. This process plays a crucial role in maintaining homeostasis, immune regulation, and the pathogenesis of PD. Apoptosis is primarily mediated through three pathways: the death receptor pathway, the mitochondrial pathway, and the endoplasmic reticulum stress pathway.

#### 3.2.1 Death receptor pathway

Also known as the extrinsic pathway, the death receptor pathway is initiated by extracellular signals. In PD, the Fas/Fas ligand (FasL) system triggers apoptosis by forming a death-inducing signaling complex (DISC), which activates caspase-8 (6). Activated caspase-8 can directly cleave and activate the executioner caspase-3. Additionally, it engages the mitochondrial pathway by cleaving Bid, a pro-apoptotic Bcl-2 family member, leading to mitochondrial outer membrane permeabilization (MOMP), cytochrome c (Cyt c) release, and subsequent activation of caspase-9. This cross-talk synergistically mediates the extrinsic apoptosis of dopaminergic neurons (7, 8).

#### 3.2.2 Mitochondrial pathway

Mitochondria serve as a central regulator of intrinsic apoptosis. In PD, mitochondrial dysfunction is an early and pivotal event driving dopaminergic neuronal loss. Key features include a decline in mitochondrial membrane potential and increased permeability, facilitating the release of pro-apoptotic factors such as Cyt c. In the cytosol, Cyt c, Apaf-1, and dATP form the apoptosome, which activates caspase-9, thereby initiating a proteolytic cascade involving effector caspases like caspase-3 (9). This cascade is closely linked to severe impairment of mitochondrial complex I, which disrupts oxidative phosphorylation (OXPHOS), leading to ATP depletion, elevated reactive oxygen species (ROS) production, and diminished glutathione (GSH) levels. ATP depletion further disrupts mitochondrial calcium (Ca^2+^) buffering, leading to neuronal Ca^2+^ dyshomeostasis. This activates nitric oxide synthase (NOS), increasing nitric oxide (NO) production. NO reacts with superoxide anion (O₂•^−^) to form peroxynitrite (ONOO^−^), exacerbating oxidative damage and ultimately inducing apoptosis (10).

#### 3.2.3 Endoplasmic reticulum pathway

The endoplasmic reticulum (ER) is essential for protein folding, processing, and intracellular Ca^2+^ homeostasis. Pathological insults such as misfolded protein accumulation or Ca^2+^ imbalance disrupt ER function, triggering ER stress. While the adaptive unfolded protein response (UPR) initially aims to restore proteostasis, persistent or excessive ER stress in PD switches the cellular outcome to PCD, promoting dopaminergic neuron apoptosis (11, 12).

##### 3.2.3.1 α-syn and tyrosine hydroxylase (TH)

The abnormal aggregation of α-syn, a core pathological hallmark of PD, disrupts ER homeostasis, inducing persistent ER stress and activating apoptotic pathways (13). Concurrently, ER stress can upregulate TH expression, leading to overactivation of dopaminergic neurons and the generation of neurotoxic metabolites, thereby exacerbating apoptosis (14–17).

##### 3.2.3.2 CHOP protein

CHOP (C/EBP homologous protein) is a key transcription factor mediating ER stress-induced apoptosis. Under sustained ER stress, CHOP is upregulated via UPR branches such as PERK/eIF2α/ATF4 and IRE1/XBP1. Elevated CHOP disrupts apoptotic homeostasis by downregulating anti-apoptotic proteins (e.g., Bcl-2) and upregulating pro-apoptotic proteins (e.g., Bim), while simultaneously impairing the adaptive UPR, collectively driving dopaminergic neuron apoptosis in PD (18).

Moreover, neurotrophic factors such as brain-derived neurotrophic factor (BDNF) and glial cell line-derived neurotrophic factor (GDNF) promote dopaminergic neuron survival, synaptogenesis, and repair in PD. They exert these protective effects partly by activating the cAMP-CREB signaling pathway and regulating downstream gene transcription. GDNF and BDNF can also alleviate mitochondrial dysfunction and apoptosis induced by neurotoxins like MPTP and 6-OHDA, highlighting their potential as therapeutic targets (19, 20).

In conclusion, apoptosis plays a significant role in PD progression, although its intricate regulatory networks warrant further investigation.

### 3.3 Pyroptosis

Pyroptosis is a highly pro-inflammatory form of PCD characterized by cell swelling, plasma membrane pore formation, and the release of cellular contents. While crucial for host defense against pathogens, its excessive activation can trigger sustained neuroinflammation and has been demonstrated to play a key role in the onset and progression of PD (21).

The execution of pyroptosis primarily relies on two core pathways: the classical caspase-1-dependent pathway and the non-classical caspase-4/5/11-dependent pathway. Additionally, activated caspase-3 can cleave gasdermin E (GSDME), linking pyroptosis to apoptosis. Notably, the cellular level of GSDME determines the fate following caspase-3 activation: high expression promotes pyroptosis, whereas low expression leads to apoptosis (22).

Given that pyroptosis is inherently driven by inflammasome activation and a robust inflammatory response, and considering that neuroinflammation is a major driver of PD pathogenesis (23), the role of pyroptosis in PD has attracted significant attention. Several mechanisms have been elucidated. For instance, dopamine can modulate NLRP3-dependent inflammation via the dopamine D1 receptor pathway. Yan et al. (24) demonstrated that exogenous stimuli activate Toll-like receptors (TLRs) and the downstream nuclear factor-κB (NF-κB) pathway, which upregulates NLRP3 expression. Concurrently, disruptions in intracellular ATP and K^+^ homeostasis elevate reactive oxygen species (ROS) levels, damage mitochondrial DNA (mtDNA), and promote the recruitment of NIMA-related kinase 7 (NEK7) to bind and activate the NLRP3 inflammasome. This cascade ultimately cleaves gasdermin D (GSDMD), executing pyroptosis and significantly accelerating PD progression (22, 25). Furthermore, in PD models, GSDMD has been shown to promote glial cell activation and dopaminergic neuronal death; accordingly, inhibiting GSDMD effectively alleviates PD symptoms (22). Clinical studies corroborate these findings, showing that serum levels of pyroptosis-related pro-inflammatory factors are significantly elevated in PD patients (26).

In summary, pyroptosis is closely linked to PD pathogenesis by driving neuroinflammation, and its interaction with apoptotic pathways may further accelerate disease progression.

### 3.4 Necroptosis

In the pathological context of PD, necroptosis represents a receptor-mediated PCD pathway that can be triggered by death receptors such as tumor necrosis factor receptor 1 (TNFR1). TNFR1 activation leads to the engagement of receptor-interacting protein kinase 1 (RIPK1) (27). Subsequently, under conditions of caspase-8 inhibition, deubiquitinated RIPK1 interacts with and recruits RIPK3 via its receptor-interacting protein homology (RHIM) domain. RIPK3 then recruits the mixed lineage kinase domain-like protein (MLKL). The assembly of RIPK1, RIPK3, and MLKL forms the necrosome (complex IIb), thereby initiating necroptosis (28, 29). Within this complex, RIPK3 undergoes autophosphorylation and subsequently phosphorylates MLKL, prompting MLKL to form membrane-disrupting oligomers. This ultimately compromises plasma membrane integrity, leading to lytic cell death (30).

The critical role of this pathway in PD models has been confirmed. Callizot et al. (9) found that neurotoxin treatment significantly upregulates TNF-*α* levels alongside enhanced phosphorylation of RIPK1, RIPK3, and MLKL—hallmarks of activated necroptosis. In contrast, Oñate et al. (6) introduced the concept of “necroptotic axonal degeneration,” demonstrating that inhibiting RIPK3 or MLKL effectively delays dopaminergic neuronal degeneration and ameliorates PD-related motor dysfunction (28). Studies indicate that RIPK1, by activating the downstream RIPK3-MLKL cascade, not only induces neuronal death but also modulates neuroinflammation in glial cells, creating a vicious cycle that accelerates neurodegeneration (9, 31). Mitochondrial reactive oxygen species (ROS) act as a key regulator, promoting RIPK1 autophosphorylation, which in turn enhances necrosome assembly and MLKL pore-forming capacity, ultimately driving necroptosis (32).

Furthermore, α-syn, a core pathological protein in PD, has been closely linked to necroptosis (33). Although the precise molecular mechanisms remain to be fully elucidated, existing evidence strongly suggests a significant pathological interplay between them, providing a crucial theoretical foundation for further exploration of PD neurodegenerative mechanisms.

### 3.5 Ferroptosis

Accumulating evidence confirms that dysregulation of iron metabolism plays a central role in the pathogenesis of PD. Elevated iron levels in the substantia nigra are closely correlated with disease progression, the severity of neurodegeneration, and motor deficits (34).

Ferroptosis is a form of PCD driven by iron-dependent lipid peroxidation, where reactive species derived from free iron catalyze the generation of lipid hydroperoxides. This cell death process hinges on three core mechanisms: (1) accumulation of intracellular labile iron, (2) failure of the glutathione (GSH)/glutathione peroxidase 4 (GPX4) antioxidant axis, often via inhibition of System Xc^−^ (the cystine/glutamate antiporter), and (3) peroxidation of membrane polyunsaturated fatty acids (PUFAs) (35).

While iron is essential as a redox cofactor for cellular metabolism and ATP production in the central nervous system (CNS), neural tissues are particularly vulnerable to oxidative damage caused by iron overload and compromised antioxidant defenses (36). In PD, systemic iron accumulation contributes to striatal dopamine depletion, loss of neuromelanin, and the formation of intracellular Lewy bodies, which are primarily composed of aggregated α-syn. Concurrently, depletion of antioxidant enzymes within the glutathione system exacerbates oxidative stress, lipid peroxidation, and mitochondrial dysfunction (36).

Research indicates that ferroptosis-specific inhibitors and iron chelators can effectively prevent dopaminergic neuronal death. Dionísio et al. (37) demonstrated that α-syn aggregation induces mitochondrial oxidative stress and lipid peroxidation in an iron-dependent manner, thereby activating ferroptosis. Furthermore, the transcription factor Nrf2, a master regulator of cellular antioxidant responses and iron/lipid metabolism, counteracts 6-hydroxydopamine-induced ferroptosis upon activation, highlighting its therapeutic potential (38, 39).

In summary, these findings collectively establish ferroptosis as a pivotal molecular event in PD neurodegeneration. Therefore, therapeutic strategies aimed at bolstering antioxidant pathways, inhibiting lipid peroxidation, modulating the System Xc^−^–GPX4/GSH axis, and activating the Nrf2 pathway hold promise for mitigating PD pathology (see Table 1).

**Table 1:** Mechanism of PCD in PD.

Classical forms of PCD	Core process	Key molecular events	Mechanistic role in PD	Therapeutic strategies and targets
Autophagy (3–5)	Maintains intracellular homeostasis by clearing abnormal protein aggregates and damaged organelles.	1. Core Pathways: Autophagy-lysosome pathway, Mitophagy. 2. Regulatory Genes: LRRK2, GBA, PINK1, PRKN, DJ-1, APOE4. 3. Bidirectional Regulation: Bcl2-BECN1 complex, Rab1.	1. Impaired autophagy is an early key event in PD, hindering α-syn clearance and promoting its aggregation. 2. Forms a vicious bidirectional regulatory cycle with *α*-syn.	Enhancing Autophagy (3–5): Target the autophagy-lysosome pathway and mitophagy to promote α-syn clearance.
Apoptosis	A non-inflammatory form of PCD that precisely responds to intra- and extracellular signals.	1. Death Receptor Pathway (6–8): Fas/FasL, Caspase-8. 2. Mitochondrial Pathway (9, 10): Cytochrome c (Cyt c), Apaf-1, Caspase-9. 3. ER Stress Pathway (11–20): CHOP, Bcl-2 family.	1. All three pathways contribute to the orderly loss of dopaminergic neurons. 2. Mitochondrial dysfunction is an initiating factor; ER stress is triggered by α-syn aggregation.	Inhibiting Apoptosis: 1. Target the Bcl-2 family, caspases, and mitochondria. 2. using neurotrophic factors (e.g., BDNF, GDNF) to activate pro-survival pathways such as the cAMP-CREB axis.
Pyroptosis	A pro-inflammatory, lytic cell death characterized by cell swelling, membrane pore formation, and massive release of inflammatory factors.	1. Executioner Proteins (22): GSDMD, GSDME. 2. Inflammasome (23, 24): NLRP3. 3. Pro-inflammatory Factors (22–26): IL-1β, IL-18.	1. Drives neuroinflammation, creating a positive feedback loop that exacerbates dopaminergic neuron death. 2. Cross-talks with apoptosis (via Caspase-3).	Inhibiting Pyroptosis (22–26): Target the NLRP3 inflammasome and GSDMD to alleviate neuroinflammation.
Necroptosis	A receptor-mediated, pro-inflammatory form of PCD resulting in plasma membrane rupture and release of cellular contents.	1. Core Complex (9, 27–29): Necrosome (RIPK1-RIPK3-MLKL). 2. Initiating Signal (27): TNFR1, TNF-α. 3. Key Regulation (28, 29): Loss of Caspase-8 activity.	1. Activated when apoptosis is inhibited, leading to lytic death and exacerbated inflammation. 2. Closely associated with abnormally aggregated α-syn and mitochondrial ROS.	Inhibiting Necroptosis (27–29): Target the kinase activity of RIPK1, RIPK3, and MLKL.
Ferroptosis	An iron-dependent, regulated cell death driven by lipid peroxidation.	1. Core Events (35): Labile iron accumulation, GSH depletion, GPX4 inactivation, lipid peroxidation. 2. Key Regulation (36–39): System Xc^−^, Nrf2 pathway.	1. Dysregulated iron metabolism in the substantia nigra is a core feature of PD. 2. Leads to oxidative stress and mitochondrial dysfunction; α-syn can activate ferroptosis.	Inhibiting Ferroptosis (35–39): Employ antioxidants, activate the Nrf2 pathway, and replenish GSH.

## 4 Research progress on acupuncture regulation of PCD for PD prevention and treatment

### 4.1 Acupuncture prevents and treats PD by regulating autophagy

#### 4.1.1 PINK1/Parkin signaling pathway

Mitochondria, as double-membrane cytoplasmic organelles, are integral to cellular processes including energy production and calcium homeostasis. Mitochondrial dysfunction is a key contributor to PD, and mitophagy represents the selective autophagic clearance of damaged mitochondria (40). Research has established the PINK1/Parkin pathway as central to mitochondrial quality control (41). Impairment of this pathway leads to the accumulation of dysfunctional mitochondria, resulting in excessive reactive oxygen species (ROS) production and the triggering of apoptotic cascades.

Electroacupuncture at the Yanglingquan (GB34) and Taichong (LR3) acupoints has been shown to activate the PINK1/Parkin-mediated mitophagy pathway in the hippocampus, thereby suppressing the expression of reactive oxygen species (ROS) and interleukin-1β (IL-1β). This intervention promotes the degradation of abnormally aggregated α-syn, mitigates dopaminergic neuronal damage, and ultimately ameliorates motor dysfunction associated with PD (42).

Studies have demonstrated that acupuncture can alleviate PD pathological symptoms by modulating mitophagy via the PINK1/Parkin pathway. Haiyang et al. (43) reported that a combined regimen of acupuncture (at Baihui [GV20], Fengfu [GV16], Dazhui [GV14], Jinjin [EX-HN12], Jinsuo [GV8], Mingmen [GV4] and Yaoyangguan [BL31]) and Chinese herbal medicine restored mitophagy levels via the PINK1/Parkin and Nix/BNIP3L pathways, thereby improving behavioral performance and alleviating symptoms in PD mice.

Further supporting this, Zhang et al. (44) found that electroacupuncture at Fengfu (GV16), Taichong (LR3), and Zusanli (ST36) modulated the SIRT3/PINK1/Parkin axis, thereby rectifying imbalances in mitophagy, promoting the clearance of damaged mitochondria, restoring mitochondrial function, and ameliorating PD symptoms.

Another study (45) in male PD rats showed that electroacupuncture at the same acupoint set (Taichong, Fengfu, and Zusanli) elevated mRNA expression of PINK1, Parkin, Beclin-1, and LC3 while reducing expression of α-syn and p62. This intervention activated mitophagy, enhanced tyrosine hydroxylase (TH) expression in the substantia nigra, and ultimately improved motor dysfunction in MPTP-induced PD mice.

Furthermore, abnormal autophagy has been observed in the enteric nervous system of PD patients (46). Electroacupuncture at the Tianshu (ST25) point can alleviate PD-associated constipation by enhancing the autophagic state within the enteric nervous system, protecting enteric neuronal function, and regulating intestinal motility.

In summary, acupuncture appears to alleviate PD symptoms, in part, by activating mitophagy via the PINK1/Parkin signaling pathway. This helps maintain mitochondrial homeostasis and exerts neuroprotective effects, highlighting a potential mechanism for its therapeutic action.

#### 4.1.2 mTOR signaling pathway

Research has demonstrated that the mechanistic target of rapamycin (mTOR) plays a crucial role in regulating neuronal function and autophagy, serving as a central hub for cell proliferation and survival implicated in various stages of PD (47). In PD, the phosphorylation level of Akt—a positive regulator of mTOR—is reduced, while the negative regulator AMP-activated protein kinase (AMPK) is activated. This dual alteration attenuates mTOR activity, which in turn inhibits downstream protein synthesis mediated by eukaryotic translation initiation factor 4E-binding protein 1 (4E-BP1) and ribosomal protein S6 kinase (p70S6K). As this synthesis is essential for long-term neuronal survival, its inhibition ultimately exacerbates PD-related neuronal damage (47).

Studies indicate that acupuncture can exert a dual therapeutic effect by specifically activating the mTOR signaling pathway (48). It not only effectively suppresses abnormally heightened autophagy in PD model rats, thereby correcting autophagic imbalance and associated neuronal injury, but also promotes the repair of damaged synaptic structures and enhances synaptic function. These findings underscore the central mediating role of the mTOR pathway in acupuncture-induced regulation of autophagy and synaptic repair.

Tian et al. (49) reported that electroacupuncture at the Yanglingquan (GB34) acupoint ameliorated PD pathology in model mice, primarily by enhancing the expression of lysosomal membrane proteins and increasing lysosomal enzyme activity. This enhancement restored impaired autophagy-lysosomal fusion and degradation capacity, leading to reduced α-syn aggregation and the recovery of dopaminergic neuronal function. Notably, this therapeutic effect was independent of mTOR-mediated regulation of autophagy initiation. These findings suggest a heterogeneous role for mTOR pathway-induced autophagy in PD, the specific mechanisms of which warrant further investigation.

#### 4.1.3 Additional regulatory pathways

Beyond the core PINK1/Parkin and mTOR pathways, acupuncture modulates autophagy through other key signaling cascades. Research indicates that electroacupuncture at Baihui (GV20) and Taichong (LR3) enhances autophagic flux by activating the Nrf2 pathway, evidenced by upregulated markers (Atg7, LC3II) and reduced p62 levels (50). This mitigates MPTP-induced dopaminergic neuron loss and α-syn pathology, thereby improving motor function in PD mice.

Separately, electroacupuncture at bilateral Sanyinjiao (SP6) and Neiguan (PC6) points was found to regulate the KIF5A/Miro1 axis, reducing autophagosome accumulation in the prefrontal cortex, improving mitochondrial morphology, and lowering ROS levels. These changes ameliorated cognitive dysfunction and neurological damage in PD models (51).

In summary, acupuncture employs a multi-pathway strategy to regulate autophagy in PD: it activates the Nrf2 pathway to bolster general autophagic capacity and antioxidant response; modulates the KIF5A/Miro1 pathway to facilitate mitochondrial trafficking and mitophagic clearance; alongside its established roles via PINK1/Parkin and mTOR signaling. These coordinated actions converge to reduce α-syn aggregation, mitigate oxidative stress, and restore neuronal homeostasis, although the precise interplay among these pathways warrants further mechanistic delineation.

### 4.2 Acupuncture prevents PD by modulating apoptosis

A study demonstrated that electroacupuncture at Fengfu (GV16) and Taichong (LR3) acupoints exerts neuroprotective effects in 6-hydroxydopamine (6-OHDA)-induced PD model rats through dual regulation of the endoplasmic reticulum stress (ERS) pathway. Specifically, treatment upregulates the molecular chaperone GRP78 to enhance protein-folding capacity and promote adaptation, while concurrently inhibiting the pro-apoptotic PERK/eIF2α/CHOP axis and downstream activation of caspase-12 and caspase-3. This reduces dopaminergic neuronal apoptosis and improves motor function (52).

Further supporting this mechanism, Ma et al. (53) reported that the same electroacupuncture regimen in a rotenone-induced PD model facilitates the clearance of abnormally aggregated, misfolded α-syn in the substantia nigra. This alleviates ERS and downregulates the transcriptional activity of key unfolded protein response (UPR) factors, including ATF6 and XBP-1, leading to improved motor function (53).

Moreover, in the 1-methyl-4-phenylpyridinium (MPP^+^) model, electroacupuncture suppresses apomorphine-induced rotational behavior and abnormal spontaneous activity. This protective effect is associated with the activation of pro-survival signaling pathways, specifically Akt and brain-derived neurotrophic factor (BDNF), within the substantia nigra (54).

In conclusion, the core mechanism by which acupuncture prevents and treats PD lies in protecting dopaminergic neurons and delaying their apoptotic process. Although direct investigations into acupuncture’s regulation of apoptosis for PD intervention remain insufficient, future studies are urgently needed to elucidate the synergistic interactions and intrinsic connections among its multifaceted mechanisms of action. Future research could target the endoplasmic reticulum stress pathway by, on one hand, upregulating the expression of the molecular chaperone GRP78 to enhance protein-folding capacity and promote cellular adaptation; and on the other hand, inhibiting the PERK/eIF2α/CHOP apoptotic signaling axis and the subsequent activation of caspase-12 and caspase-3, thereby reducing dopaminergic neuronal apoptosis and improving motor function in PD models (55).

### 4.3 Acupuncture alleviates PD by modulating pyroptosis

A growing body of evidence confirms that NLRP3 inflammasome-mediated, GSDMD-dependent pyroptosis plays a pivotal role in PD progression, suggesting that inhibition of this pathway may alleviate PD symptoms (56).

Electroacupuncture at specific acupoints can exert neuroprotection in PD by concurrently targeting anti-oxidative and anti-pyroptotic pathways. Research indicates that stimulation of Fengfu (GV16), Taichong (LR3), and Zusanli (ST36) acupoints activates the Nrf2 signaling pathway while inhibiting the NLRP3/caspase-1/GSDMD-mediated pyroptosis cascade. This dual regulation downregulates key inflammatory cytokines, including IL-1β and IL-18, in both the substantia nigra and systemic circulation, thereby mitigating neuroinflammation and pyroptotic cell death (57).

In a related mechanism focusing on the gut-brain axis, Guo et al. (58) reported that electroacupuncture improves behavioral deficits and motor dysfunction in MPTP-induced PD mice. This therapeutic effect is associated with enhanced intestinal barrier function, achieved through modulation of the intestinal NLRP3 inflammasome, highlighting a novel pathway through which acupuncture may confer systemic neuroprotection. Similarly, Quan et al. (59) reported that EA suppressed LPS-mediated NLRP3 inflammasome activation in the substantia nigra, downregulating the expression of NLRP3, caspase-1, and the downstream pro-inflammatory cytokine IL-1β, thereby attenuating neuroinflammation and dopaminergic neuronal damage. Furthermore, research indicates that EA can upregulate Sirt3 expression in the substantia nigra, which inhibits NLRP3 inflammasome activation and IL-1β release while reducing GSDMD cleavage. This dual action decreases pyroptotic cell death, ultimately mitigating dopaminergic neuron loss and improving motor dysfunction in PD rats (60).

Additionally, elevated expression of toll-like receptors (TLRs) is observed in the brains of PD patients, where they co-localize with α-syn in Lewy bodies and trigger inflammatory responses (56). Therefore, inhibiting TLR signaling represents a novel therapeutic strategy for PD by modulating neuroinflammation (61).

### 4.4 Acupuncture alleviates PD by modulating necroptosis

The understanding of acupuncture’s role in modulating PD pathogenesis has evolved from a holistic view to molecular mechanistic insights. Research demonstrates that necroptosis, mediated by the RIPK1/RIPK3/MLKL pathway, is significantly activated in PD models (62). This activation is associated with elevated mitochondrial reactive oxygen species (mtROS), which can trigger the mitochondrial localization and pore-forming activity of GSDMD, thereby facilitating a transition from apoptotic to necroptotic cell death.

Furthermore, a close regulatory interplay exists between necroptosis and the PINK1/Parkin-mediated mitophagy pathway, the latter being crucial for clearing damaged mitochondria (41). Impairment of the PINK1/Parkin pathway disrupts mitophagy, exacerbating mitochondrial dysfunction and mtROS burst. This in turn activates RIPK1 and promotes necrosome formation, ultimately accelerating dopaminergic neuronal death.

Acupuncture is posited to exert neuroprotective effects by multi-targeted intervention in this “mitophagy-necroptosis axis.” Specifically, it enhances mitophagic activity, restores PINK1/Parkin function, clears damaged mitochondria, and reduces mtROS, thereby suppressing RIPK1 activation at its source (42).

Based on the established mechanisms by which acupuncture modulates programmed necrosis in intracerebral hemorrhage models, combined with the known roles of programmed cell death (PCD) in PD pathogenesis and the demonstrated ability of acupuncture to regulate multiple PCD forms (e.g., autophagy, apoptosis) in PD models, we hypothesize that acupuncture may also inhibit the assembly and execution of the core necroptotic pathway. This potential inhibition could involve interfering with RIPK1-RIPK3 interaction, blocking MLKL phosphorylation and oligomerization, and thereby disrupting necrosome function (63).

The anti-neuroinflammatory and antioxidant properties of acupuncture further contribute by lowering pro-inflammatory factors (e.g., TNF-*α*, IL-1β), inhibiting death receptor-mediated initiation of necroptosis, and upregulating neurotrophic factors such as BDNF (64).

In summary, acupuncture demonstrates multidimensional regulatory advantages in mitigating PD-associated neurodegeneration. It coordinately enhances mitophagic clearance, suppresses the necroptotic pathway, and modulates the inflammatory microenvironment. These insights provide a novel perspective on acupuncture’s molecular mechanisms and establish a theoretical foundation for developing disease-modifying strategies targeting the mitophagy-necroptosis interplay (65).

### 4.5 Acupuncture alleviates PD by modulating ferroptosis

Research indicates that electroacupuncture at the Fengfu (GV16) and Baihui (GV20) acupoints can activate the Nrf2/GPX4 signaling pathway. This activation enhances the antioxidant capacity of dopaminergic neurons and inhibits iron-dependent lipid peroxidation, thereby reducing neuronal loss, improving motor dysfunction, and ultimately delaying PD progression (66).

Guided by the oxidative stress hypothesis of PD etiology, Lee et al. (67) investigated the therapeutic potential of acupuncture at the Yanglingquan (GB34) acupoint in MPTP-induced mouse models. Their findings demonstrated that this intervention effectively modulates the striatal antioxidant system, enhancing endogenous antioxidant capacity. Consequently, it significantly inhibits dopaminergic neuronal death in the nigrostriatal pathway and restores striatal levels of DJ-1 protein alongside the activities of key antioxidant enzymes, superoxide dismutase (SOD) and catalase (CAT).

Further supporting this, Huang et al. (68) demonstrated that acupuncture, via activation of the Nrf2/ARE signaling pathway, effectively scavenges excess reactive oxygen species (ROS), reduces levels of lipid peroxidation products (e.g., malondialdehyde, MDA; 4-hydroxynonenal, 4-HNE), and boosts glutathione reserves. These actions alleviate oxidative stress-mediated neuronal damage and apoptosis, contributing to improved cognitive and motor functions in PD models.

Moreover, acupuncture’s regulation of the Nrf2/ARE pathway exhibits multi-target synergistic characteristics. It integrates mechanisms such as neuroinflammation and autophagy modulation to form a comprehensive neuroprotective network with antioxidant, anti-inflammatory, and anti-apoptotic effects.

Although direct investigations into acupuncture’s regulation of ferroptosis remain relatively limited, accumulating evidence suggests that it can indirectly influence key ferroptotic pathways through multi-targeted and multi-pathway synergistic effects, playing a beneficial role in PD prevention and treatment.

However, further studies are warranted to mechanistically clarify the causal relationship and delineate the specific molecular pathways linking acupuncture to the regulation of ferroptosis (see Table 2).

**Table 2:** Research on acupuncture regulation of PCD for PD prevention and treatment.

Classical forms of PCD	Researcher(s)	Acupuncture mode	Acupoints (code)	Stimulation parameters	Induction	Proposed mechanism of action	Primary outcome
Autophagy	Hsu et al. (42)	Electroacupuncture	Yanglingquan (GB34), Taichong (LR3)	50 Hz, 1 mA, 20 min/day, 5 days	MPTP (i.p.)	Activates the PINK1/Parkin-mediated mitophagy pathway in the hippocampus; inhibits ROS and IL-1β expression.	Promotes degradation of abnormal α-syn aggregates, reduces dopaminergic neuronal damage, and improves motor dysfunction.
	Haiyang et al. (43)	Manual acupuncture	Baihui (GV20), Fengfu (GV16), Dazhui (GV14), Jinjin (EX-HN12), Jinsuo (GV8), Mingmen (GV4), Yaoyangguan (BL31)	20 min/day, 6 weeks	MPTP (i.p.)	Restores mitophagy levels via the PINK1/Parkin and Nix/BNIP3L pathways.	Improves behavioral scores and alleviates PD symptoms in mice.
	Zhang et al. (44)	Electroacupuncture	Fengfu (GV16), Taichong (LR3), Zusanli (ST36)	2 Hz, 1 mA, 20 min/day, 12 days	MPTP (i.p.)	Modulates the SIRT3/PINK1/Parkin pathway, rectifying imbalanced mitophagy and promoting clearance of damaged mitochondria.	Ameliorates PD symptoms.
	Zhang et al. (45)	Electroacupuncture	Taichong (LR3), Fengfu (GV16), Zusanli (ST36)	100 Hz, 3 mA, 20 min/day, 5 days	MPTP (i.p.)	Upregulates PINK1, Parkin, Beclin-1, and LC3 mRNA; downregulates α-syn and p62 mRNA, activating mitophagy.	Improves motor dysfunction and enhances tyrosine hydroxylase (TH) expression in the substantia nigra.
	Song et al. (46)	Electroacupuncture	Tianshu (ST25)	2/15 Hz (alternating), 2 mA, 20 min/day, 5 days/week, 4 weeks	Rotenone (s.c.)	Improves autophagic status in the enteric nervous system, protecting enteric neuronal function and regulating motility.	Alleviates PD-associated constipation.
	Ning et al. (48)	Manual acupuncture	Baihui (GV20), Yintang (EX-HN3), Hegu (LI4), Taichong (LR3), Mingmen (GV4)	20 min/day, 28 days	6-OHDA (medial forebrain bundle)	Inhibits excessive autophagy and corrects autophagic balance; improves synaptic function.	Exerts bidirectional regulatory effects on PD pathology.
	Tian et al. (49)	Electroacupuncture	Yanglingquan (GB34)	10 min/day (with needle manipulation for 15 s every 5 min)	MPTP (i.p.)	Enhances lysosomal membrane protein expression and enzyme activity, restoring autophagosome-lysosome fusion and degradation.	Reduces α-syn aggregation and restores dopaminergic neuronal function.
	Zhang et al. (50)	Electroacupuncture	Baihui (GV20), Taichong (LR3)	2 Hz, 20 min/day, 14 days	MPTP (i.p.)	Activates the Nrf2 pathway, upregulating Atg7 and LC3II while reducing p62, thereby enhancing autophagic flux.	Mitigates dopaminergic neuron loss and abnormal α-syn expression, improving motor dysfunction.
	Li et al. (51)	Electroacupuncture	Sanyinjiao (SP6), Neiguan (PC6)	5/25 Hz (alternating), 0.5 mA, 20 min/day, 6 days/week, 30 days	6-OHDA (medial forebrain bundle)	Modulates the KIF5A/Miro1 pathway, reducing autophagosome number, improving mitochondrial morphology, and decreasing ROS.	Improves cognitive dysfunction and neurological damage.
Apoptosis	Ma et al. (52)	Electroacupuncture	Fengfu (GV16), Taichong (LR3)	2 Hz, 1 mA, 20 min/day (7, 14, 21, or 28 days)	Rotenone (s.c.)	1. Upregulates chaperone GRP78 to enhance ER protein folding. 2. Inhibits the PERK/eIF2α/CHOP axis and downstream caspases-12/3.	Reduces dopaminergic neuron apoptosis and improves motor function.
	Ma et al. (53)	Electroacupuncture	Fengfu (GV16), Taichong (LR3)	2 Hz, 1 mA, 20 min/day (7, 14, 21, or 28 days)	Rotenone (s.c.)	Clears misfolded α-syn aggregates in the substantia nigra, reduces ER stress, and downregulates ATF6 & XBP-1.	Ameliorates motor bradykinesia.
	Lin et al. (54)	Electroacupuncture	Changqiang (GV1), Taichong (LR3)	50 Hz, 1 mA, 150 μs pulse width, 20 min	MPTP (i.p.)	Inhibits apomorphine-induced rotation; activates Akt and BDNF pathways in the substantia nigra.	Exerts neuroprotective effects.
Pyroptosis	Zhang et al. (56)	Electroacupuncture	Fengfu (GV16), Taichong (LR3), Zusanli (ST36)	2 Hz, 1 mA, 30 min/day, 2 weeks	Rotenone (oral gavage)	Activates Nrf2 and inhibits the NLRP3/caspase-1/GSDMD pathway, downregulating IL-1β and IL-18.	Alleviates neuroinflammation and pyroptosis, conferring neuroprotection.
	Guo et al. (58)	Electroacupuncture	Fengfu (GV16), Taichong (LR3), Zusanli (ST36)	2 Hz, 1 mA, 15 min/day, 7 days	MPTP (i.p.)	Modulates the intestinal NLRP3 inflammasome, enhancing gut barrier function.	Improves behavioral deficits and motor dysfunction.
	Quan et al. (59)	Electroacupuncture	Sishencong (EX-HN1), Sanyinjiao (SP6)	2/10 Hz (alternating), 30 min/day, 4 weeks	6-OHDA (medial forebrain bundle)	Inhibits LPS-mediated NLRP3 activation and downregulates NLRP3, caspase-1, and IL-1β in the substantia nigra.	Reduces neuroinflammation and dopaminergic neuron damage.
	Wang et al. (60)	Electroacupuncture	Fengfu (GV16), Taichong (LR3), Zusanli (ST36)	2 Hz, 1 mA, 30 min/day, 4 weeks	Rotenone (in oil, s.c.)	Upregulates Sirt3, inhibits NLRP3/IL-1β axis, and reduces GSDMD cleavage.	Attenuates dopaminergic neuron loss and improves motor dysfunction.
	Hsu et al. (42)	Electroacupuncture	Yanglingquan (GB34), Taichong (LR3)	50 Hz, 1 mA, 20 min/day, 5 days	MPTP (i.p.)	Activates hippocampal PINK1/Parkin-mediated mitophagy; inhibits ROS and IL-1β.	Promotes α-syn degradation, reduces neuronal damage, and improves motor function.
	Haiyang et al. (43)	Manual acupuncture	Baihui (GV20), Fengfu (GV16), Dazhui (GV14), Jinjin (EX-HN12), Jinsuo (GV8), Mingmen (GV4), Yaoyangguan (BL31)	20 min/day, 6 weeks	MPTP (i.p.)	Restores mitophagy via PINK1/Parkin and Nix/BNIP3L pathways.	Improves behavioral scores and PD symptoms.
	Zhang et al. (44)	Electroacupuncture	Fengfu (GV16), Taichong (LR3), Zusanli (ST36)	2 Hz, 1 mA, 20 min/day, 12 days	MPTP (i.p.)	Modulates SIRT3/PINK1/Parkin to correct mitophagy, clearing damaged mitochondria.	Ameliorates PD symptoms.
	Zhang et al. (45)	Electroacupuncture	Taichong (LR3), Fengfu (GV16), Zusanli (ST36)	100 Hz, 3 mA, 20 min/day, 5 days	MPTP (i.p.)	Upregulates PINK1, Parkin, Beclin-1, LC3 mRNA; downregulates α-syn, p62 mRNA.	Improves motor dysfunction and enhances nigral TH expression.
	Wang et al. (66)	Electroacupuncture	Fengfu (GV16), Baihui (GV20)	5 Hz, 1 mA, 30 min/day, 12 days	MPTP (i.p.)	Activates Nrf2/GPX4, enhancing antioxidant capacity and inhibiting iron-dependent lipid peroxidation.	Inhibits death-receptor-mediated necroptosis, reduces neuron loss, and improves motor function.
	Lee et al. (67)	Electroacupuncture	Yanglingquan (GB34)	60 s needling/day, 12 days	MPTP (i.p.)	Modulates the striatal antioxidant system and boosts endogenous antioxidants.	Inhibits death-receptor-mediated necroptosis initiation.
Ferroptosis	Wang et al. (66)	Electroacupuncture	Fengfu (GV16), Baihui (GV20)	5 Hz, 1 mA, 30 min/day, 12 days	MPTP (i.p.)	Activates Nrf2/GPX4, enhancing antioxidant capacity and inhibiting iron-dependent lipid peroxidation.	Reduces dopaminergic neuron loss, improves motor dysfunction, and delays PD progression.
	Lee et al. (67)	Manual acupuncture	Yanglingquan (GB34)	60 s needling/day, 12 days	MPTP (i.p.)	Modulates the striatal antioxidant system and boosts endogenous antioxidants.	Suppresses dopaminergic neuron death in the nigrostriatal pathway and restores striatal DJ-1, SOD, and CAT activity.

## 5 Summary and perspectives

PD is a neurodegenerative disorder closely associated with aging, whose progression is intricately linked to dysregulated PCD. The classical forms of PCD—including autophagy, apoptosis, pyroptosis, necroptosis, and ferroptosis—do not operate in isolation within PD pathology. Instead, they engage in extensive crosstalk, mutually regulating one another to collectively form the core pathological basis for dopaminergic neuron loss and abnormal α-syn aggregation.

Acupuncture is widely recognized as a non-pharmacological therapeutic strategy for PD. It exerts synergistic, multi-targeted effects on key PCD pathways by: inhibiting excessive apoptosis, maintaining autophagic homeostasis, blocking the inflammatory cascade associated with pyroptosis, mitigating iron-dependent lipid peroxidation, reducing neuronal loss, and enhancing blood–brain barrier integrity. These actions underscore the “bidirectional modulation” and “multi-target integration” that characterize acupuncture’s regulatory effects. By intervening at nodal points within the interconnected PCD network, acupuncture helps maintain a dynamic equilibrium between cell survival and death in the brain. This provides a robust rationale for its application in neurodegenerative diseases and enriches the contemporary biological understanding of acupuncture-mediated neuroprotection (69, 70). Furthermore, acupuncture demonstrates promising synergistic potential when integrated with other therapies within a multidisciplinary framework, particularly for modulating autophagy, pyroptosis, and ferroptosis in PD (43).

Although numerous studies support the beneficial role of acupuncture in modulating PCD, the evidence base remains predominantly preclinical, with a significant translational gap to clinical practice. Current research is constrained by methodological limitations, including small sample sizes, inadequate control of confounding variables, and substantial heterogeneity in outcome measures, which collectively compromise the reliability and generalizability of findings. Further challenges stem from a lack of treatment standardization. Variations in acupoint selection, stimulation parameters (e.g., frequency, waveform, duration), and treatment courses hinder the comparability and reproducibility of results across studies. Additionally, the inherent difficulty in designing rigorous control interventions—such as credible sham acupuncture that mimics the sensory experience without eliciting specific therapeutic effects—poses a persistent methodological challenge that may introduce bias.

Future studies should urgently focus on elucidating the spatiotemporal dynamics of different PCD forms in PD progression. It is essential to systematically evaluate how specific acupuncture parameters—such as technique, stimulation frequency, duration, and treatment course—affect the PCD network. Promoting large-scale, multicenter randomized controlled trials will be crucial to validate these mechanistic insights and translate them into precise, feasible, and effective intervention strategies for PD.

## References

[1] Bloem BR Okun MS Klein C. Parkinson's disease. Lancet (London, England). (2021) 397:2284–303. doi: 10.1016/S0140-6736(21)00218-X, 33848468

[2] Xiao B Zhou Z Chao Y Tan EK. Pathogenesis of Parkinson's disease. Neurol Clin. (2025) 43:185–207. doi: 10.1016/j.ncl.2024.12.003, 40185518

[3] Lizama BN Chu CT. Neuronal autophagy and mitophagy in Parkinson's disease. Mol Asp Med. (2021) 82:100972. doi: 10.1016/j.mam.2021.100972, 34130867 PMC8665948

[4] Nechushtai L Frenkel D Pinkas-Kramarski R. Autophagy in Parkinson's disease. Biomolecules. (2023) 13:435. doi: 10.3390/biom13101435, 37892117 PMC10604695

[5] Lv QK Tao KX Wang XB Yao XY Pang MZ Liu JY . Role of α-synuclein in microglia: autophagy and phagocytosis balance neuroinflammation in Parkinson's disease. Inflammation Res. (2023) 72:443–62. doi: 10.1007/s00011-022-01676-x, 36598534

[6] Oñate M Catenaccio A Salvadores N Saquel C Martinez A Moreno-Gonzalez I . Correction: the necroptosis machinery mediates axonal degeneration in a model of Parkinson disease. Cell Death Differ. (2020) 27:2294. doi: 10.1038/s41418-020-0507-2, 32047275 PMC7410843

[7] Gao J Zhao Y Wang C Ji H Yu J Liu C . A novel synthetic chitosan selenate (CS) induces apoptosis in A549 lung cancer cells via the Fas/FasL pathway. Int J Biol Macromol. (2020) 158:689–97. doi: 10.1016/j.ijbiomac.2020.05.016 32387597

[8] Liu W Vetreno RP Crews FT. Hippocampal TNF-death receptors, caspase cell death cascades, and IL-8 in alcohol use disorder. Mol Psychiatry. (2021) 26:2254–62. doi: 10.1038/s41380-020-0698-4, 32139808 PMC7483234

[9] Callizot N Combes M Henriques A Poindron P. Necrosis, apoptosis, necroptosis, three modes of action of dopaminergic neuron neurotoxins. PLoS One. (2019) 14:e0215277. doi: 10.1371/journal.pone.0215277, 31022188 PMC6483187

[10] Yang W Hao W Meng Z Ding S Li X Zhang T . Molecular regulatory mechanism and toxicology of neurodegenerative processes in MPTP/probenecid-induced progressive Parkinson's disease mice model revealed by transcriptome. Mol Neurobiol. (2021) 58:603–16. doi: 10.1007/s12035-020-02128-5, 32997292 PMC7843579

[11] Awasthi A Maparu K Singh S. Ferroptosis role in complexity of cell death: unrevealing mechanisms in Parkinson's disease and therapeutic approaches. Inflammopharmacology. (2025) 33:1271–87. doi: 10.1007/s10787-025-01672-7, 39998712

[12] Mahoney-Sánchez L Bouchaoui H Ayton S Devos D Duce JA Devedjian JC. Ferroptosis and its potential role in the physiopathology of Parkinson's disease. Prog Neurobiol. (2021) 196:101890. doi: 10.1016/j.pneurobio.2020.101890, 32726602

[13] Forloni G. Alpha synuclein: neurodegeneration and inflammation. Int J Mol Sci. (2023) 24:914. doi: 10.3390/ijms24065914, 36982988 PMC10059798

[14] Picca A Guerra F Calvani R Romano R Coelho-Júnior HJ Bucci C . Mitochondrial dysfunction, protein misfolding and neuroinflammation in Parkinson's disease: roads to biomarker discovery. Biomolecules. (2021) 11:508. doi: 10.3390/biom11101508, 34680141 PMC8534011

[15] Li Y Liu Z Wang D Gao H Zhu Z Wang Y . Ucf-101 protects in vivoandin vitro models of PD against 6-hydroxydopamine toxicity by alleviating endoplasmic reticulum stress via the Wnt/β-catenin pathway. J Clin Neurosci. (2020) 71:217–25. doi: 10.1016/j.jocn.2019.11.023, 31883812

[16] Kim DY Shin JY Lee JE Kim HN Chung SJ Yoo HS . A selective ER-phagy exerts neuroprotective effects via modulation of α-synuclein clearance in parkinsonian models. Proc Natl Acad Sci USA. (2023) 120:e2221929120. doi: 10.1073/pnas.2221929120, 37669380 PMC10500278

[17] Gopinath A Mackie P Hashimi B Buchanan AM Smith AR Bouchard R . DAT and TH expression marks human Parkinson's disease in peripheral immune cells. NPJ Parkinsons Dis. (2022) 8:72. doi: 10.1038/s41531-022-00333-8, 35672374 PMC9174333

[18] Pazi MB Belan DV Komarova EY Ekimova IV. Intranasal administration of GRP78 protein (HSPA5) confers neuroprotection in a lactacystin-induced rat model of Parkinson's disease. Int J Mol Sci. (2024) 25:951. doi: 10.3390/ijms25073951, 38612761 PMC11011682

[19] Pak ME Ahn SM Jung DH Lee HJ Ha KT Shin HK . Electroacupuncture therapy ameliorates motor dysfunction via brain-derived neurotrophic factor and glial cell line-derived neurotrophic factor in a mouse model of Parkinson's disease. J Gerontol A Biol Sci Med Sci. (2020) 75:712–21. doi: 10.1093/gerona/glz256 31644786

[20] Er S Airavaara M. Protective mechanisms by glial cell line-derived neurotrophic factor and cerebral dopamine neurotrophic factor against the α-synuclein accumulation in Parkinson's disease. Biochem Soc Trans. (2023) 51:245–57. doi: 10.1042/BST20220770, 36794783

[21] Ma H Zhu Y Zhao X Jiang L Yang J Yang T . Pyroptosis: inflammatory cell death mechanism and its pathological roles in neurological diseases and injuries. Apoptosis. (2025) 30:2057–76. doi: 10.1007/s10495-025-02160-7, 40813542

[22] Zhang X Zhang Y Wang B Xie C Wang J Fang R . Pyroptosis-mediator GSDMD promotes Parkinson's disease pathology via microglial activation and dopaminergic neuronal death. Brain Behav Immun. (2024) 119:129–45. doi: 10.1016/j.bbi.2024.03.038, 38552923

[23] Huang P Zhang Z Zhang P Feng J Xie J Zheng Y . TREM2 deficiency aggravates NLRP3 Inflammasome activation and Pyroptosis in MPTP-induced Parkinson's disease mice and LPS-induced BV2 cells. Mol Neurobiol. (2024) 61:2590–605. doi: 10.1007/s12035-023-03713-0, 37917301 PMC11043123

[24] Yan Y Jiang W Liu L Wang X Ding C Tian Z . Dopamine controls systemic inflammation through inhibition of NLRP3 inflammasome. Cell. (2015) 160:62–73. doi: 10.1016/j.cell.2014.11.047, 25594175

[25] Wang S Yuan YH Chen NH Wang HB. The mechanisms of NLRP3 inflammasome/pyroptosis activation and their role in Parkinson's disease. Int Immunopharmacol. (2019) 67:458–64. doi: 10.1016/j.intimp.2018.12.019, 30594776

[26] Clarke N Thornton P Reader V Lindsay N Digby Z Mullen B . Anti-Neuroinflammatory and anti-inflammatory effects of the NLRP3 inhibitor NT-0796 in subjects with Parkinson's disease. Mov Disord. (2025) 40:2199–208. doi: 10.1002/mds.30307, 40792655 PMC12553988

[27] Shao L Yu S Ji W Li H Gao Y. The contribution of necroptosis in neurodegenerative diseases. Neurochem Res. (2017) 42:2117–26. doi: 10.1007/s11064-017-2249-1 28382594

[28] Park JS Leem YH Kim DY Park JM Kim SE Kim HS. Neuroprotective and anti-inflammatory effects of the RIPK3 inhibitor GSK872 in an MPTP-induced mouse model of Parkinson's disease. Neurochem Int. (2024) 181:105896. doi: 10.1016/j.neuint.2024.105896 39491747

[29] Gupta R Kumari S Tripathi R Ambasta RK Kumar P. Unwinding the modalities of necrosome activation and necroptosis machinery in neurological diseases. Ageing Res Rev. (2023) 86:101855. doi: 10.1016/j.arr.2023.101855, 36681250

[30] Xiang H. The interplay between α-synuclein aggregation and necroptosis in Parkinson's disease: a spatiotemporal perspective. Front Neurosci. (2025) 19:1567445. doi: 10.3389/fnins.2025.1567445, 40264913 PMC12011736

[31] Kang A Qiao Y Pan S Yan F Chen H Bai Y. From RIPK1 to necroptosis: pathogenic mechanisms in neurodegenerative diseases. Neurochem Res. (2025) 50:194. doi: 10.1007/s11064-025-04448-1, 40493155

[32] Zhou Y Cai Z Zhai Y Yu J He Q He Y . Necroptosis inhibitors: mechanisms of action and therapeutic potential. Apoptosis. (2024) 29:22–44. doi: 10.1007/s10495-023-01905-6, 38001341

[33] Liu Z Kang K Shan S Wang S Li X Yong H . Chronic carbon disulfide exposure induces parkinsonian pathology via α-synuclein aggregation and necrosome complex interaction. iScience. (2023) 26:107787. doi: 10.1016/j.isci.2023.107787, 37731606 PMC10507234

[34] Ding XS Gao L Han Z Eleuteri S Shi W Shen Y . Ferroptosis in Parkinson's disease: molecular mechanisms and therapeutic potential. Ageing Res Rev. (2023) 91:102077. doi: 10.1016/j.arr.2023.102077, 37742785

[35] Yan HF Zou T Tuo QZ Xu S Li H Belaidi AA . Ferroptosis: mechanisms and links with diseases. Signal Transduct Target Ther. (2021) 6:49. doi: 10.1038/s41392-020-00428-9, 33536413 PMC7858612

[36] Wang X Wang Z Cao J Dong Y Chen Y. Ferroptosis mechanisms involved in hippocampal-related diseases. Int J Mol Sci. (2021) 22:902. doi: 10.3390/ijms22189902, 34576065 PMC8472822

[37] Dionísio PA Amaral JD Rodrigues CMP. Oxidative stress and regulated cell death in Parkinson's disease. Ageing Res Rev. (2021) 67:101263. doi: 10.1016/j.arr.2021.101263, 33540042

[38] Zhou M Xu K Ge J Luo X Wu M Wang N . Targeting Ferroptosis in Parkinson's disease: mechanisms and emerging therapeutic strategies. Int J Mol Sci. (2024) 25:42. doi: 10.3390/ijms252313042, 39684753 PMC11641825

[39] Ko CJ Gao SL Lin TK Chu PY Lin HY. Ferroptosis as a major factor and therapeutic target for neuroinflammation in Parkinson's disease. Biomedicine. (2021) 9:1679. doi: 10.3390/biomedicines9111679 PMC8615560 34829907

[40] Liang H Ma Z Zhong W Liu J Sugimoto K Chen H. Regulation of mitophagy and mitochondrial function: natural compounds as potential therapeutic strategies for Parkinson's disease. Phytother Res. (2024) 38:1838–62. doi: 10.1002/ptr.8156, 38356178

[41] Malpartida AB Williamson M Narendra DP Wade-Martins R Ryan BJ. Mitochondrial dysfunction and Mitophagy in Parkinson's disease: from mechanism to therapy. Trends Biochem Sci. (2021) 46:329–43. doi: 10.1016/j.tibs.2020.11.007 33323315

[42] Hsu WT Chen YH Yang HB Lin JG Hung SY. Electroacupuncture improves motor symptoms of Parkinson's disease and promotes neuronal autophagy activity in mouse brain. Am J Chin Med. (2020) 48:1651–69. doi: 10.1142/S0192415X20500822 33202151

[43] Haiyang WU Ying W Wei H Huihui LI Haisheng JI Xiuxiu L. Protective effect of Tongdu Tiaoshen acupuncture combined with Xiaoxuming decoction on dopaminergic neurons in Parkinson's disease model. J Trad Chinese Med. (2023) 43:484–93. doi: 10.19852/j.cnki.jtcm.20230214.005, 37147749 PMC10133951

[44] Zhang GJ Wang Y Li JL Ma J Wang YC. Effects of electroacupuncture on mitophagy mediated by SIRT3/PINK1/Parkin pathway in Parkinson's disease mice. Zhen Ci Yan Jiu. (2024) 49:221–30. doi: 10.13702/j.1000-0607.20230654, 38500318

[45] Zhang GJ Jin TT Lv YC Wang Y Ma J Wang YC. Effects of Electroacupuncture on mitochondrial autophagy in the substantia Nigra of 1-Methyl-4-phenyl-1,2,3,6-tetrahydropyridine-induced mouse models. Chin J Rehabil Med. (2026) 1–10.

[46] Song LZ Xu N Yu Z Yang H Xu CC Qiu Z . The effect of electroacupuncture at ST25 on Parkinson's disease constipation through regulation of autophagy in the enteric nervous system. Anatom Rec. (2023) 306:3214–28. doi: 10.1002/ar.25148 36655864

[47] Zhu Z Yang C Iyaswamy A Krishnamoorthi S Sreenivasmurthy SG Liu J . Balancing mTOR signaling and autophagy in the treatment of Parkinson's disease. Int J Mol Sci. (2019) 20:28. doi: 10.3390/ijms20030728, 30744070 PMC6387269

[48] Ning B Wang Z Wu Q Deng Q Yang Q Gao J . Acupuncture inhibits autophagy and repairs synapses by activating the mTOR pathway in Parkinson's disease depression model rats. Brain Res. (2023) 1808:148320. doi: 10.1016/j.brainres.2023.148320, 36914042

[49] Tian T Sun YH Wu HG Pei J Zhang J Zhang Y . Acupuncture promotes mTOR-independent autophagic clearance of aggregation-prone proteins in mouse brain. Sci Rep. (2016) 6:19714. doi: 10.1038/srep19714 26792101 PMC4726430

[50] Zhang J Fu Z Wen F Lyu P Huang S Cai X . Electroacupuncture ameliorated locomotor symptoms in MPTP-induced mice model of Parkinson's disease by regulating autophagy via Nrf2 signaling. J Neurophysiol. (2025) 133:490–501. doi: 10.1152/jn.00497.2024 39745671

[51] Li MZ Chen JF Chen MX Li HY Zhang JY Gao D . Effects of electroacupuncture on cognitive impairment and mitophagy mediated by KIF5A/Miro1pathway in Parkinson's disease mice. Chin Acupunct Moxibustion. (2025) 45:1111–9. doi: 10.13703/j.0255-2930.20250227-k0004 40825695

[52] Ma J Yuan L Wang SJ Lei J Wang Y Li YN . Effects of electroacupuncture at “Fengfu” and “Taichong” on the expression of related proteins of endo-plasmic reticulum stress in Parkinson’s disease rats [Thesis]. Chin J Rehabil Med. (2019) 34:772–7. https://www.globethesis.com/?t=2334330545983187

[53] Ma J Yuan L Wang SJ Lei J Wang Y Li YN . Electroacupuncture improved locomotor function by regulating expression of tyrosine hydroxylase and α-synuclein proteins and transcription activating factor 6 and transcription factor X box binding protein 1 mRNAs in substantia nigra of rats with Parkinson's disease. Zhen Ci Yan Jiu. (2019) 44:805–9. doi: 10.13702/j.1000-0607.180600, 31777229

[54] Lin JG Chen CJ Yang HB Chen YH Hung SY. Electroacupuncture promotes recovery of motor function and reduces dopaminergic neuron degeneration in rodent models of Parkinson's disease. Int J Mol Sci. (2017) 18:846. doi: 10.3390/ijms18091846, 28837077 PMC5618495

[55] Motawi TK Al-Kady RH Abdelraouf SM Senousy MA. Empagliflozin alleviates endoplasmic reticulum stress and augments autophagy in rotenone-induced Parkinson's disease in rats: targeting the GRP78/PERK/eIF2α/CHOP pathway and miR-211-5p. Chem Biol Interact. (2022) 362:110002. doi: 10.1016/j.cbi.2022.110002, 35654124

[56] Cheng Y Tong Q Yuan Y Song X Jiang W Wang Y . α-Synuclein induces prodromal symptoms of Parkinson's disease via activating TLR2/MyD88/NF-κB pathway in Schwann cells of vagus nerve in a rat model. J Neuroinflammation. (2023) 20:36. doi: 10.1186/s12974-023-02720-1, 36788559 PMC9926693

[57] Zhang XL Hu MN Rong Z Li YN Wang Y Ma J. Effect of electroacupuncture on Nrf2/NLRP3/Caspase-1 pathway mediated-pyroptosis in mice with Parkinson's disease. Zhen Ci Yan Jiu. (2024) 49:15–22. doi: 10.13702/j.1000-0607.20230407 38239134

[58] Guo L Hu H Jiang N Yang H Sun X Xia H . Electroacupuncture blocked motor dysfunction and gut barrier damage by modulating intestinal NLRP3 inflammasome in MPTP-induced Parkinson's disease mice. Heliyon. (2024) 10:e30819. doi: 10.1016/j.heliyon.2024.e30819, 38774094 PMC11107113

[59] Quan J Liu X Liang S Nie L Zhang L Hong X . Electroacupuncture suppresses motor impairments via microbiota-metabolized LPS/NLRP3 signaling in 6-OHDA induced Parkinson's disease rats. Int Immunopharmacol. (2025) 162:115089. doi: 10.1016/j.intimp.2025.115089, 40836410

[60] Wang Y Wang YC Ma J. Effects of electroacupuncture on Sirt3/NLRP3/GSDMD signaling pathway in the substantia nigra of midbrain of rats with Parkinson's disease. Zhen Ci Yan Jiu. (2024) 49:384–90. doi: 10.13702/j.1000-0607.20230024 38649206

[61] Liu YY Guo YB Zhai HY Lei DB Wang H Zhao SC . Effect of electroacupuncture regulating NLRP3/Caspase-1 pathway on pyroptosis of dopaminergic neurons in rats with Parkinson's disease. Zhen Ci Yan Jiu. (2022) 47:983–92. doi: 10.13702/j.1000-0607.20211016 36453675

[62] Weindel CG Martinez EL Zhao X Mabry CJ Bell SL Vail KJ . Mitochondrial ROS promotes susceptibility to infection via gasdermin D-mediated necroptosis. Cell. (2022) 185:e3223:3214–31. doi: 10.1016/j.cell.2022.06.038 PMC9531054 35907404

[63] Cai GF Sun ZR Zhuang Z Zhou HC Gao S Liu K . Cross electro-nape-acupuncture ameliorates cerebral hemorrhage-induced brain damage by inhibiting necroptosis. World J Clin Cases. (2020) 8:1848–58. doi: 10.12998/wjcc.v8.i10.1848, 32518774 PMC7262720

[64] Xin YY Wang JX Xu AJ. Electroacupuncture ameliorates neuroinflammation in animal models. Acupunct Med. (2022) 40:474–83. doi: 10.1177/09645284221076515, 35229660

[65] Lin QS Chen P Wang WX Lin CC Zhou Y Yu LH . RIP1/RIP3/MLKL mediates dopaminergic neuron necroptosis in a mouse model of Parkinson disease. Lab Investig. (2020) 100:503–11. doi: 10.1038/s41374-019-0319-5, 31506635

[66] Wang M Zheng HS Ye WL Mao JD Zhang K Yang L . The mechanism of electroacupuncture-mediated improvement in Parkinson's disease by inhibiting ferroptosis through activating the Nrf2/GPX4 signal pathway. Front Aging Neurosci. (2025) 17:1551404. doi: 10.3389/fnagi.2025.1551404, 40400910 PMC12092470

[67] Lee Y Choi G Jeon H Kim D Ryu S Koo S . Acupuncture stimulation at GB34 suppresses 1-methyl-4-phenyl-1,2,3,6-tetrahydropyridine-induced oxidative stress in the striatum of mice. J Physiol Sci. (2018) 68:455–62. doi: 10.1007/s12576-017-0547-7, 28601951 PMC10717018

[68] Huang TI Hsieh CL. Effects of acupuncture on oxidative stress amelioration via Nrf2/ARE-related pathways in Alzheimer and Parkinson diseases. Evid Based Complement Alternat Med. (2021) 2021:6624976. doi: 10.1155/2021/6624976, 33995547 PMC8096560

[69] Yuan GM Su FY Li PY Guo YM Zhang J Xu Y . Cerebral protective effect of acupuncture based on its regulative effect on programmed cell death. Zhen Ci Yan Jiu. (2025) 50:210–6. doi: 10.13702/j.1000-0607.20230934, 40059056

[70] Ko JH Lee H Kim SN Park HJ. Does acupuncture protect dopamine neurons in Parkinson's disease rodent model? A systematic review and meta-analysis. Front Aging Neurosci. (2019) 11:102. doi: 10.3389/fnagi.2019.00102, 31139074 PMC6517785

